# METTL3‐mediated m6A modification enhances lncRNA H19 stability to promote endothelial cell inflammation and pyroptosis to aggravate atherosclerosis

**DOI:** 10.1096/fj.202401337RR

**Published:** 2024-10-21

**Authors:** Feng Tang, Long‐hai Tian, Xiao‐han Zhu, Sen Yang, Huan Zeng, Yong‐yao Yang

**Affiliations:** ^1^ Department of Cardiology The Second People's Hospital of Guiyang Guiyang Guizhou China; ^2^ Department of Cardiology Guizhou Provincial People's Hospital Guiyang Guizhou China

**Keywords:** atherosclerosis, lncRNA H19, m6A modification, METTL3, vascular disease

## Abstract

This study explored the impact of N^6^‐methyladenosine (m6A) modification on the regulation of long noncoding RNA (lncRNA) and atherosclerosis progression. An atherosclerosis cell model was established by treating human aortic endothelial cells (HAECs) with oxidized low‐density lipoprotein. Additionally, an atherosclerotic animal model was developed using ApoE^−/−^ C57BL/6 male mice fed a high‐fat diet. Both models were employed to assess the expression changes of proteins associated with m6A modification. First, the effect of m6A modification writer protein methyltransferase‐like 3 (METTL3) knockdown on changes in the level of pyroptosis in HAECs was investigated, and bioinformatic analysis confirmed that lncRNA H19 (H19) was the potential target of m6A modification. RNA‐binding protein immunoprecipitation assays were subsequently performed to explore the interaction between H19 and the m6A writer protein METTL3, as well as the reader protein recombinant insulin‐like growth factor 2 mRNA‐binding protein 2 (IGF2BP2). Finally, the effect of H19 expression on pyroptosis levels in HAECs was evaluated. In the aortas of atherosclerosis mice, overall m6A levels were significantly elevated compared with controls (*p* < .05), with METTL3 and METTL14 mRNA and protein levels notably increased (*p* < .05). Similarly, ox‐LDL‐treated HAECs showed a significant rise in m6A levels, along with increased METTL3 and METTL14 expression (*p* < .05). METTL3 knockdown in HAECs led to decreased pyroptosis, as evidenced by reduced lactate dehydrogenase release and lower levels of IL‐1β, IL‐18, and IL‐6 (*p* < .05). Overexpression of H19 reversed these effects, indicating METTL3's role in promoting atherosclerosis by stabilizing H19 through m6A modification. H19 was the primary target lncRNA molecule of METTL3‐mediated m6A modification in the pathogenesis of atherosclerosis. METTL3‐mediated m6A modification regulated H19 expression, thereby aggravating atherosclerosis by activating pyroptosis.

## Graphical abstract

Figure S1METTL3 expression in the aorta of mice with atherosclerosis (AS).A.Relative mRNA expression levels of METTL3 in control, AS, AS+siNC, and AS+si‐METTL3 groups; B. Representative Western blot images and quantification of METTL3 protein levels, normalized to β‐actin, across the same groups. **P  < 0.01 vs. Control group; ##P < 0.01 vs. AS+siNC group.

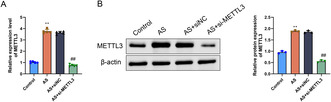

**Abbreviations**
ELISA: enzyme‐linked immunosorbent assay
HAECs: human aortic endothelial cells
HDL‐C: high‐density lipoprotein cholesterol
HRP: Anti‐Rabbit IgG H&L
ICAM‐1: intercellular cell adhesion molecule 1
LDH: lactate dehydrogenase
LDL‐C: low‐density lipoprotein cholesterol
lncRNA: long noncoding RNA
m6A: N^6^‐methyladenosine
METTL3: methyltransferase‐like 3
METTL14: methyltransferase‐like 14
miRNA: microRNA
mRNA: messenger RNA
MTC: methyltransferase complex
OD: optical density
ox‐LDL: oxidized low‐density lipoprotein
PVDF: polyvinylidene fluoride
RIP: RNA‐binding protein immunoprecipitation
si‐METTL3: METTL3 siRNA
siNC: control siRNA
STAT1: signal transducer and activator of transcription 1
TC: total cholesterol
TG: triglycerides
VCAM‐1: vascular cell adhesion molecule 1
WTAP: Wilms' tumor 1 associating protein

## 1 INTRODUCTION

Atherosclerosis is a chronic progressive vascular disease characterized by the gradual accumulation of lipids, collagen, cholesterol, and other substances within the arterial wall, leading to the formation of plaque. These plaque structures contribute to clinical syndromes such as vascular stenosis and dysfunction, ultimately hindering normal blood flow.^1^ In modern society, diets high in fat and sugar, sedentary lifestyles, and habits such as smoking and alcoholism^2^ have significantly increased the prevalence of atherosclerosis over the past 10 years. According to the American Heart Association, over 40% of adults worldwide suffer from varying degrees of atherosclerosis.^3^ Furthermore, because atherosclerosis is a precursor to severe cerebrovascular and cardiovascular conditions, including myocardial infarction, ischemic stroke, and peripheral arterial disease,^4^ it represents a serious hidden health threat. The onset and progression of atherosclerosis result from the complex interplay of various pathophysiological processes, including arterial endothelial injury, neovascularization, oxidative stress, inflammatory immune responses, lipid metabolism disorders, and abnormal cell proliferation and apoptosis.^1^ A significant correlation between pyroptosis and atherosclerosis progression was recently reported, with the dysfunction and demise of endothelial cells marking the initiation of this cardiovascular condition.^5^ Pyroptosis is a form of programmed cell death characterized by the release of pro‐inflammatory cytokines and the formation of pores in the cell membrane, often involving the activation of caspase‐1.^6^ This process contributes to inflammation and is linked to various diseases, including atherosclerosis.^7^ Inflammatory cytokines are instrumental in inducing endothelial cell pyroptosis within the context of atherosclerosis. This, in turn, triggers the release of additional inflammatory cytokines, attracting monocytes and furthering atherosclerosis progression.^8^ The mechanisms driving pyroptosis in endothelial cells during atherosclerosis are multifaceted, involving multiple regulatory pathways. However, the lack of comprehensive research elucidating the specific molecular processes involved has impeded the development of effective therapeutic strategies for atherosclerosis. Therefore, further exploration of the pyroptosis mechanism in atherosclerosis will contribute to identifying novel treatment targets, developing more effective drugs, formulating more precise preventive strategies, and alleviating the economic burden and threats to health caused by cerebrovascular and cardiovascular diseases.

Long noncoding RNA (lncRNA) refers to RNA molecules longer than 200 nucleotides that, while lacking protein‐coding function, play crucial roles in regulating gene expression, cellular behavior, and signaling pathways through various mechanisms.^9^ The primary functions of lncRNA included as follows: (1) serving as a precursor of short functional RNA, (2) regulating protein activity, (3) regulating transcription, translation, and chromatin modification, and (4) acting as a microRNA (miRNA) sponge and binding miRNA to affect the expression of target genes through specific competition.^10^ The correlation between lncRNA and atherosclerosis has gradually become a research focus in recent years. Several lncRNAs, including STEEL, LASSIE, SENCR, LEENE, MALAT1, and H19, have been implicated in the development and progression of atherosclerosis through their involvement in biological processes such as inflammatory response, cholesterol metabolism, and pyroptosis.^11^ Additionally, many lncRNAs act as signaling molecules that modulate pyroptosis, particularly in endothelial cells.^12^, ^13^ A study summarizes the role of lncRNAs in atherosclerosis by highlighting their involvement in regulating endothelial cell function, modulating inflammatory responses, interacting with miRNAs, and discussing their potential applications in the diagnosis and treatment of atherosclerotic diseases^14^ Pan et al. specifically demonstrated how the lncRNA H19 promotes atherosclerosis by influencing inflammatory signaling pathways in vascular smooth muscle cells.^15^ Overall, lncRNAs are integral to the pathological progression of atherosclerosis, underscoring the importance of further exploration into the specific mechanisms by which lncRNAs regulate the onset and progression of atherosclerosis.

N^6^‐methyladenosine (m6A) modification is the most common and abundant form of post‐transcriptional RNA modification in eukaryotes, characterized by methylation at the N^6^‐position of adenosine.^16^ This modification widely exists in mRNA, miRNA, lncRNA, and other RNA molecules, affecting the expression of target genes by regulating RNA stability, crosscutting, translation, and transport.^17^ The m6A modification process is catalyzed by a methyltransferase complex (MTC), with methyltransferase‐like 3 (METTL3) serving as the core catalyst component^18^ and methyltransferase‐like 14 (METTL14), which plays a structural role and forms a stable complex with METTL3.^18^ Wilms' tumor 1 associating protein (WTAP) acts as an adaptor protein and recruits the MTC to specific RNA targets.^19^ As the significance of m6A modification has become more widely recognized, its relationship with atherosclerosis has garnered considerable research interest. Zhang et al. demonstrated that m6A modification shortened the processing time of pri‐miR‐19a into mature miR‐19a. The mature miR‐19a promoted the proliferation and migration of vascular endothelial cells, thereby promoting atherosclerosis progression.^20^ Similarly, Liu et al. claimed that m6A modification notably increased the stability of signal transducer and activator of transcription 1 (STAT1) mRNA, which enhanced the promotion of M1 macrophage polarization in blood vessels, leading to an exacerbated local inflammatory response and worsening of atherosclerosis.^21^ While most studies have focused on the influence of m6A modification on mRNA or miRNA, there has been limited investigation into the impact of m6A modification on relevant lncRNAs during atherosclerosis progression.

This study was designed address the findings and gaps in previous research. We analyzed the levels of m6A‐related writer proteins in cell and animal models of atherosclerosis and identified lncRNA H19 (H19) as a primary target of m6A modification. We then explored the effects and potential mechanisms of METTL3‐mediated m6A modification in promoting atherosclerosis progression through both in vivo and in vitro experiments. Our findings provide new insights into the role of m6A modification in atherosclerosis progression and identify potential new targets for clinical treatment.

## 2 MATERIALS AND METHODS

### 2.1 Cell culture and transfection

We used human aortic endothelial cells (HAECs) in cell models and human monocyte cell line THP‐1 for monocyte adhesion experiments. The above two cell lines were purchased from the American Type Culture Collection (Manassas, Virginia, USA) and cultured under standard conditions (5% CO_2_, 37°C, 95% humidity). HAECs were cultured in Dulbecco Modified Eagle Medium (DMEM; Servicebio, Wuhan, China) supplemented with 10% fetal bovine serum (FBS; Beyotime, Shanghai, China) and 1% penicillin/streptomycin solution (Beyotime, Shanghai, China). THP‐1 cells were cultured in Roswell Park Memorial Institute‐1640 (RPMI‐1640; Servicebio, Wuhan, China) medium (Servicebio, Wuhan, China) supplemented with 10% FBS (Beyotime) and 1% penicillin/streptomycin solution (Beyotime, Shanghai, China).

METTL3 small interfering RNA (siRNA) (si‐METTL3), recombinant insulin‐like growth factor 2 mRNA‐binding protein 2 (IGF2BP2) siRNA (si‐IGF2BP2), corresponding negative control siRNA, pcDNA3.1‐H19 plasmid for upregulating H19 expression, and negative pcDNA3.1 plasmid without any inserted exogenous genes were all designed, constructed, and synthesized by GeneChem Co., Ltd. (Shanghai, China). In strict accordance with the steps listed in the instructions, we transfected 50 nM siRNA and corresponding plasmid into HAECs using Lipofectamine RNAiMAX Transfection Reagent (Life Technologies, USA). After transfection, the cells were incubated for 48 h and collected.

Referring to methods in a previous study,^22^ we constructed an atherosclerosis cell model by treating HAECs with 5 μg/mL oxidized low‐density lipoprotein (ox‐LDL) for 24 h to simulate the state of in vivo arterial endothelial cells under conditions of high cholesterol and high oxidative stress. Thereafter, the atherosclerosis model cells were collected for subsequent experiments.

HAECs were grouped as follows: (1) Control group: HAECs without any treatment, (2) ox‐LDL group: HAECs treated with 5 μg/mL ox‐LDL for 24 h, (3) ox‐LDL+siNC group: HAECs transfected with negative siRNA and stimulated with 5 μg/mL ox‐LDL for 24 h, (4) ox‐LDL+si‐METTL3 group: HAECs transfected with si‐METTL3 and stimulated with 5 μg/mL ox‐LDL for 24 h, (5) ox‐LDL+si‐IGF2BP2 group: HAECs transfected with si‐IGF2BP2 and stimulated with 5 μg/mL ox‐LDL for 24 h, (6) siNC+vector group: HAECs cotransfected with negative siRNA and pcDNA3.1 and stimulated with 5 μg/mL ox‐LDL for 24 h, (7) si‐METTL3+vector group: HAECs cotransfected with si‐METTL3 and empty vector and stimulated with 5 μg/mL ox‐LDL for 24 h, and (8) si‐METTL3+H19 group: HAECs cotransfected with si‐METTL3 and pcDNA3.1‐H19 and stimulated with 5 μg/mL ox‐LDL for 24 h.

### 2.2 Reverse transcription real‐time quantitative PCR (RT‐qPCR)

The treated HAECs in each group were thoroughly mixed with TRIzol Reagent (Invitrogen, Carlsbad, California, USA) and centrifuged (10 000× *g*, 10 min, 4°C) to extract the total RNA. The extracted total RNA was reverse transcribed into cDNA using a PrimeScript RT Reagent Kit (Takara Biotechnology Ltd., Shiga, Japan). Then, qPCR analysis was performed using a SYBR Premix Ex Taq Kit (Takara Biotechnology Ltd., Shiga, Japan) on a Quant Studio 6 Flex Real‐Time PCR System (Applied Biosystems, Foster City, California, USA). The expression levels of METTL3, METTL14, IGF2BP2, and H19 were calibrated according to the expression level of GAPDH (internal control). Quantification and analysis were performed using the 2^‐ΔΔCT^ method. Table 1 shows the PCR primers.

**TABLE 1:** RT‐qPCR primers of human aortic endothelial cells (HAECs).

RNA	Sequences (5′ to 3′)
METTL3	5′‐TTGTCTCCAACCTTCCGTAGT‐3′ (forward) 5′‐CCAGATCAGAGAGGTGGTGTAG‐3′ (reverse)
METTL14	5′‐AGTGCCGACAGCATTGGTG‐3′ (forward) 5′‐GGAGCAGAGGTATCATAGGAAGC‐3′ (reverse)
IGF2BP2	5′‐AGTGGAATTGCATGGGAAAATCA‐3′ (forward) 5′‐CAACGGCGGTTTCTGTGTC‐3′ (reverse)
lncRNA H19	5′‐ATCGGTGCCTCAGCGTTCGG‐3′ (forward) 5′‐CTGTCCTCGCCGTCACACCG‐3′ (reverse)
GAPDH	5′‐CATCACTGCCACCCAGAAGACTG‐3′ (forward) 5′‐ATGCCAGTGAGCTTCCCGTTCAG‐3′ (reverse)

### 2.3 Determination of overall m6A level

The treated HAECs were thoroughly mixed with TRIzol Reagent and centrifuged (10 000× *g*, 10 min, 4°C) to extract the total RNA, which was purified using an RNA Miniprep Kit. Then, strictly following the steps in the instructions, the overall m6A level in HAECs was determined using an m6A RNA Methylation Assay Kit (Abcam, Cambridge, UK). Briefly, 200 ng purified poly (A) RNA was added to each well, followed by the capture antibody solution and the detection antibody solution at appropriate dilutions. Finally, the optical density (OD) values were measured at a wavelength of 450 nm using a multifunctional microplate reader (Thermo Fisher Scientific, Waltham, Massachusetts, USA). The overall m6A level in each sample was calculated after plotting a standard curve according to the OD values of standards with known concentrations.

### 2.4 Extracellular lactate dehydrogenase enzyme activity assay

We seeded 4 × 10^5^ HAECs into six‐well cell culture plates and subjected them to various treatments. Thereafter, the cells were harvested and centrifuged (9000× *g*, 10 min, 4°C), and the resultant supernatant was collected in a fresh centrifuge tube to assess the extracellular lactate dehydrogenase (LDH) enzyme activity of the differently treated HAECs using an LDH Cytotoxicity Assay Kit (C0016; Beyotime). This assay involved the conversion of lactic acid to pyruvate by LDH, resulting in NADH production and the conversion of iodonitrotetrazolium to red formazan. The enzymatic activity of LDH was quantified by measuring the absorption peak at 490 nm using a multifunctional microplate reader.

### 2.5 Extracellular interleukin (IL)‐1β, IL‐18, and IL‐6 assays

In a manner akin to the determination of extracellular LDH enzyme activity, we seeded 4 × 10^5^ HAECs in six‐well cell culture plates and exposed the cells to various treatments. Following harvesting of the treated cells and centrifugation (9000× *g*, 10 min, 4°C), the resultant supernatant was transferred to a fresh centrifuge tube. Enzyme‐linked immunosorbent assay (ELISA) kits (MULTI SCIENCES, Hangzhou, China) were used to measure the levels of IL‐1β (EK101B, Human IL‐1β ELISA Kit), IL‐18 (EK118, Human IL‐18 ELISA Kit), and IL‐6 (EK106, Human IL‐6 ELISA Kit). These kits utilize a dual‐antibody sandwich ELISA technique whereby the target protein is captured and labeled with horseradish peroxidase‐conjugated streptavidin. The resulting colorimetric signal at 450 nm is quantified using a multifunctional microplate reader to determine the target protein concentrations.

### 2.6 Pyroptosis detection assay

Pyroptosis was assessed as previously described^23^ using a FLICA 660 Caspase‐1 Assay Kit (Amyjet, China) in conjunction with flow cytometry (BD Biosciences, San Jose, California, USA) to detect activated caspase‐1 in HAECs. Propidium iodide (Sigma‐Aldrich, St. Louis, Missouri, USA) was used. Caspase‐1 activation was evaluated via flow cytometry (excitation, 660 nm; emission, 720 nm) to quantify the number of pyroptotic cells.

### 2.7 Western blotting

Total protein was extracted from HAECs using a Total Protein Extraction Kit (Sangon, China) that contained protease and phosphatase inhibitors. Briefly, after centrifugation (10 000× *g*, 10 min, 4°C), the protein concentration was measured using a BCA Protein Assay Kit (Bio‐Rad Laboratories, USA). Next, equal amounts of each protein sample (30 μg/lane) were separated by 10% sodium dodecyl‐sulfate polyacrylamide gel electrophoresis (Servicebio), transferred to a polyvinylidene fluoride membrane (Servicebio), and blocked for 1 h at room temperature with 5% skim milk. Then, the membrane was incubated at 4°C overnight with the following primary antibodies: METTL3 (1:1000, ab195352; Abcam), METTL14 (1:1000, ab300104; Abcam), IGF2BP2 (1:2000, ab124930; Abcam), vascular cell adhesion molecule 1 (VCAM‐1; 1:2000, ab134047; Abcam), intercellular cell adhesion molecule 1 (ICAM‐1; 1:1000, ab222736; Abcam), E‐selectin (1:2000, ab185698; Abcam), NLRP3 (1:1000, ab263899; Abcam), GSDMD (1:2000, ab219800; Abcam), GSDMD‐N (1:1000, ab215203; Abcam), caspase‐1 (1:1000, ab207802; Abcam), cleaved caspase‐1 (1:1000, #4199; Cell Signaling Technology, USA), and β‐actin (1:5000, ab8227; Abcam). The next day, the membrane was washed in tris‐buffered saline with Tween and incubated with Goat Anti‐Rabbit IgG H&L (HRP) secondary antibody (1:20 000, #ab6721; Abcam) for 1 h at room temperature. Finally, the membrane was developed and then visualized using a GEL imaging system (Bio‐Rad Laboratories). The protein bands were quantified using ImageJ software (NIH, USA). The relative expression levels of all target proteins were calibrated according to the expression level of β‐actin (internal control).

### 2.8 Monocyte adhesion experiment

Monocyte adhesion was assessed as previously described.^24^ Briefly, logarithmic‐phase HAECs from each group were seeded in 35 mm confocal glass‐bottomed culture dishes (Shanghai Survey Expo Biotechnology Co., Ltd., China) at a concentration of 5 × 10^3^ cells/dish. After adding high‐glucose DMEM, the cells were incubated under standard conditions for 48 h prior to performing the monocyte adhesion experiment. First, CellTracker Green CMFDA Dye (Thermo Fisher Scientific) was added to RPMI‐1640 medium containing THP‐1 cells for fluorescent labeling at 37°C for 30 min, followed by incubation at 4°C for 10 min to complete the dyeing process. After washing and resuspension, the labeled THP‐1 cells were seeded in glass‐bottomed culture dishes at a concentration of 3 × 10^5^ cells/dish and cocultured with HAECs for 1 h at 37°C. Finally, the fluorescent image was photographed and recorded using an LSM 800 confocal microscope (Zeiss, Germany), and the number of adhered THP‐1 cells was quantified using ImageJ software.

### 2.9 M6A methylated RNA‐binding protein immunoprecipitation‐quantitative real‐time PCR (MeRIP‐qPCR) detection of H19

We used a previously reported MeRIP‐qPCR method^25^ to quantify the level of m6A‐modified H19 in HAECs. Briefly, HAECs from each treatment group were thoroughly mixed with TRIzol Reagent and centrifuged (10 000× *g*, 10 min, 4°C) to extract total RNA. The extracted RNA was purified using an RNA Miniprep Kit, with 1/10 of the purified poly (A) RNA set aside as the input control. Next, Pierce Protein A/G Magnetic Beads (Thermo Fisher Scientific) were incubated with 5 μg anti‐m6A antibody (ab284130; Abcam) or rabbit anti‐IgG antibody (ab172730; Abcam) at 4°C for 2 h with rotation. After washing, the magnetic beads coupled with the m6A antibody were mixed with the purified poly (A) RNA, and 1× immunoprecipitation buffer containing RNA enzyme inhibitor. Following protease K digestion, the methylated H19 was precipitated overnight at −80°C in 5 mg glycogen, 1/10 volume of 3M sodium acetate, and 2.5 volumes of 100% ethanol. Finally, m6A abundance was detected by qPCR and normalized to the input control.

### 2.10 RNA‐binding protein immunoprecipitation experiment

To assess whether H19 interacts with the m6A modification‐related writer protein METTL3 and reader protein IGF2BP2, we performed a RNA‐binding protein immunoprecipitation (RIP) experiment following a previously published protocol.^26^ Specifically, HAECs were lysed using RIPA Lysis Buffer (Millipore, USA) and centrifuged (10 000× *g*, 10 min, 4°C). The cell lysate was divided into four portions, three of which were incubated with magnetic beads coupled to specific antibodies, while the fourth portion was left untreated as the input control. Pierce Protein A/G Magnetic Beads were incubated with 5 μg anti‐METTL3 antibody (ab195352; Abcam), anti‐IGF2BP2 antibody (ab124930; Abcam), or rabbit anti‐IgG antibody (ab172730; Abcam) at 4°C for 2 h with rotation. After washing, the magnetic beads coupled with the corresponding antibodies were incubated overnight at 4°C with the prepared cell lysate. Following digestion of the cell lysate by protease K, the precipitated RNA was extracted using TRIzol Reagent. Finally, the H19 level was determined using RT‐qPCR and calibrated according to the input control.

### 2.11 RNA stability experiment

HAECs in the logarithmic growth phase were seeded at a density of 5 × 10^3^ cells/well in 24‐well plates containing high‐glucose DMEM. After 24 h of culture under the standard conditions, actinomycin D (5 μg/mL) was added to each well. Cells were harvested before the actinomycin D treatment and at 2, 4, and 6 h after the treatment, respectively. Then, total cellular RNA was extracted using TRIzol Reagent, and the level of residual H19 was measured using RT‐qPCR.

### 2.12 Animal experiments

All animal experiments were approved and monitored by the Animal Ethics Committee of our hospital and performed in strict accordance with the Guide for the Care and Use of Laboratory Animals to ensure animal welfare.

We used 30 SPF ApoE^−/−^ C57BL/6 male mice (Charles River Laboratory Animal Co., Ltd., Beijing, China), aged 4–6 weeks and weighing 16–24 g, in our experiments. The mice were housed in the standard animal laboratory of our hospital (22°C ± 1°C, 40%–60% humidity, 12‐h light/dark cycle). All mice had free access to food and water.

The previously mentioned si‐METTL3, negative blank control siRNA, pcDNA3.1‐H19 plasmid, and negative pcDNA3.1 plasmid without any inserted exogenous genes were packaged into lentiviruses by GeneChem Co., Ltd. (Shanghai, China).

After the mice had adapted to the animal laboratory environment, they were randomly divided into five groups as follows: (1) Control group: mice were fed conventional feed for 16 weeks, (2) atherosclerosis group: mice were fed a high‐fat diet (HFD; 0.25% cholesterol and 15% fat) for 16 weeks to establish an atherosclerosis mouse model,^27^, ^28^ (3) atherosclerosis+siNC+vector group: mice were fed a HFD for 16 weeks, and lentivirus containing negative siRNA and pcDNA3.1 (2 × 10^7^ TU/mouse) was injected into the tail vein on the 4th week, (4) atherosclerosis+si‐METTL3+vector group: mice were fed a HFD for 16 weeks, and lentivirus containing si‐METTL3 and empty vector (2 × 10^7^ TU/mouse) was injected into the tail vein on the 4th week, and (5) atherosclerosis+si‐METTL3+H19 group: mice were fed a HFD for 16 weeks, and lentivirus containing si‐METTL3 and pcDNA3.1‐H19 (2 × 10^7^ TU/mouse) was injected into the tail vein on the 4th week. No mice died during the intervention period. After the intervention, the mice were sacrificed by cervical dislocation under deep anesthesia for subsequent examination.

Venous blood samples were immediately collected from the sacrificed mice by puncture of the tail vein using a disposable sterile syringe. After centrifuging the blood samples (6000× *g*, 5 min, 4°C), the serum was collected for analysis. ELISA kits (Goldenrain Biological Technology Co., Ltd., Shanghai, China) were used to determine the levels of total cholesterol (TC), triglycerides (TG), low‐density lipoprotein cholesterol (LDL‐C), high‐density lipoprotein cholesterol (HDL‐C), IL‐1β, IL‐18, and IL‐6 in the serum samples. All tests were performed in strict accordance with the manufacturer's instructions. Each sample was tested in triplicate, and the average value was taken as the final result.

Oil red O staining was used for the quantitative evaluation of intra‐aortic plaque formation. Briefly, the thoracic aortas were carefully and gently resected from sacrificed mice using optical scissors and tweezers and fixed overnight at 4°C in 4% paraformaldehyde. The next day, the outer membrane was carefully removed, and the aorta was opened longitudinally to expose the entire lumen. Then, 6‐μm thick frozen sections were cut using a freezing microtome (Leica, Germany). After drying the sections for 20 min at ambient temperature, they were successively incubated for 5 min in 100% isopropanol and 8 min in 0.5% oil red O solution (Meilun Biotechnology Co., Ltd., China) and then rinsed in 85% isopropanol for 3 min. The sections were mounted and sealed using glycerogel. Plaque formation was observed under an EVOS optical microscope. Five randomly selected fields from each sample were photographed and recorded, and ImageJ software was to quantify the ratio of plaque to the vascular area.

Total protein from the thoracic aorta was extracted using RIPA buffer containing protease and phosphatase inhibitors. The protein levels of NLRP3, GSDMD, GSDMD‐N, caspase‐1, and cleaved caspase‐1 were detected by western blotting.

### 2.13 Statistical analysis

Numerical data were expressed as mean ± standard deviation. SPSS v22.0 software (IBM Corporation, USA) was used for statistical analyses. Shapiro–Wilk test indicated that the data of each group basically conformed to a normal distribution. Therefore, differences between two groups were analyzed using independent sample *t*‐tests. Differences among multiple groups were analyzed using a one‐way analysis of variance, and if the result was significantly different, the Tukey method was performed for pairwise comparison between groups. *p*‐values <.05 were considered statistically significant.

## 3 RESULTS

### 3.1 METTL3 and METTL14 are highly expressed in the aortas of atherosclerosis mice and ox‐LDL‐treated HAECs

**FIGURE 1:**
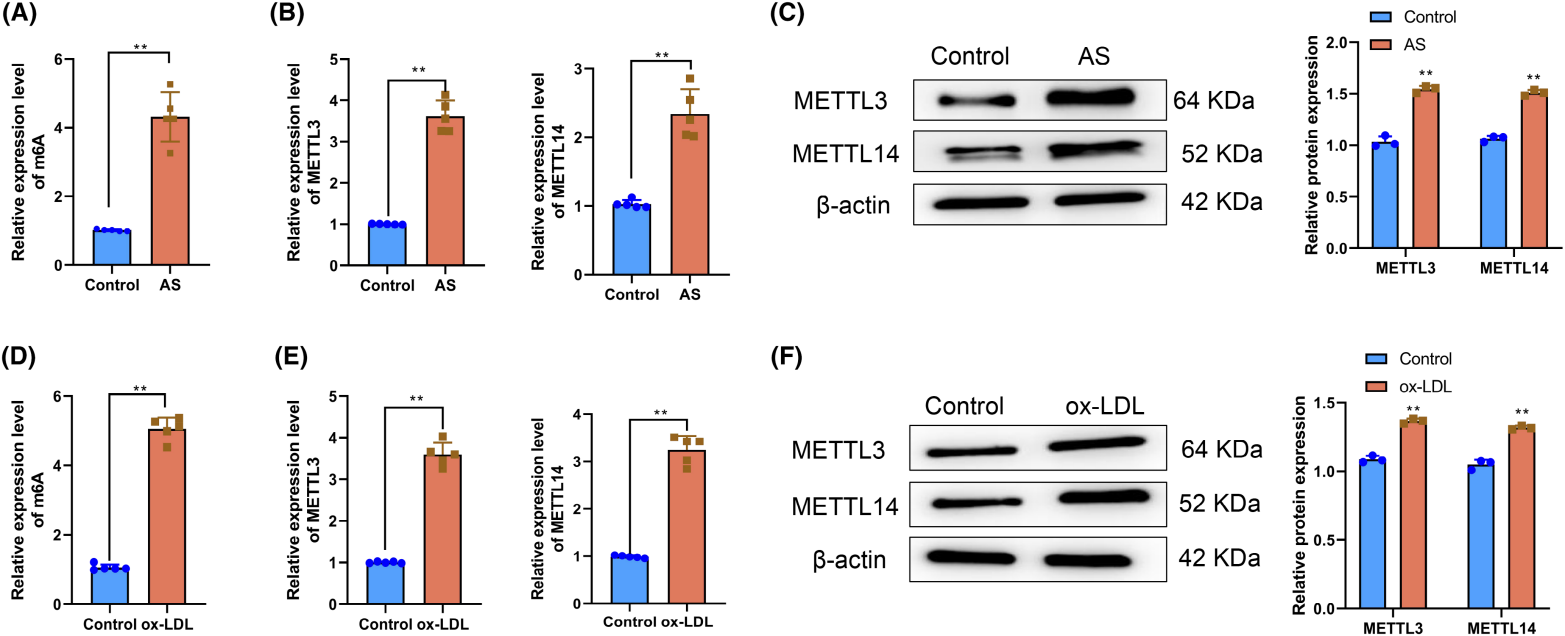
N^6^‐methyladenosine (M6A) modification‐related proteins methyltransferase‐like 3 (METTL3) and METTL14 are highly expressed in animal and cell models of AS. (A) Overall m6A level in the aortas of mice in the Control and AS groups. (B) Levels of METTL3 and METTL14 mRNA in the aortas of mice in the Control and AS groups determined using RT‐qPCR. (C) Protein levels of METTL3 and METTL14 in the aortas of mice in the Control and AS groups determined using western blotting. (D) Overall m6A level in human aortic endothelial cells (HAECs) in the Control and ox‐LDL groups. (E) Levels of METTL3 and METTL14 mRNA in HAECs in the Control and AS groups determined using RT‐qPCR. (F) Protein levels of METTL3 and METTL14 in HAECs in the Control and AS groups determined using western blotting. **p < .01 versus Control group.

First, we explored the expression of key writer proteins METTL3 and METTL14 [10] in animal and cell models of atherosclerosis during m6A modification. The overall m6A level in the aortas of mice in the atherosclerosis group was markedly increased (*p* < .05) compared with the aortas of mice in the Control group (Figure 1A). The overall m6A level in HAECs in the ox‐LDL group was significantly increased compared with HAECs in the Control group (*p* < .05) (Figure 1D). The RT‐qPCR and western blotting results showed that both mRNA and protein levels of METTL3 and METTL14 in the aortas of mice in the atherosclerosis group were notably increased compared with mRNA and protein levels of METTL3 and METTL14 in the Control group (*p* < .05) (Figure 1B,C). Similarly, both mRNA and protein levels of METTL3 and METTL14 in HAECs in the ox‐LDL group were significantly increased compared with HAECs in the Control group (*p* < .05) (Figure 1E,F). The above results indicated that m6A modification mediated by METTL3 and METTL14 might be a vital feature of atherosclerosis.

### 3.2 Changes in METTL3 expression affect the pyroptosis level of HAECs treated with ox‐LDL

**FIGURE 2:**
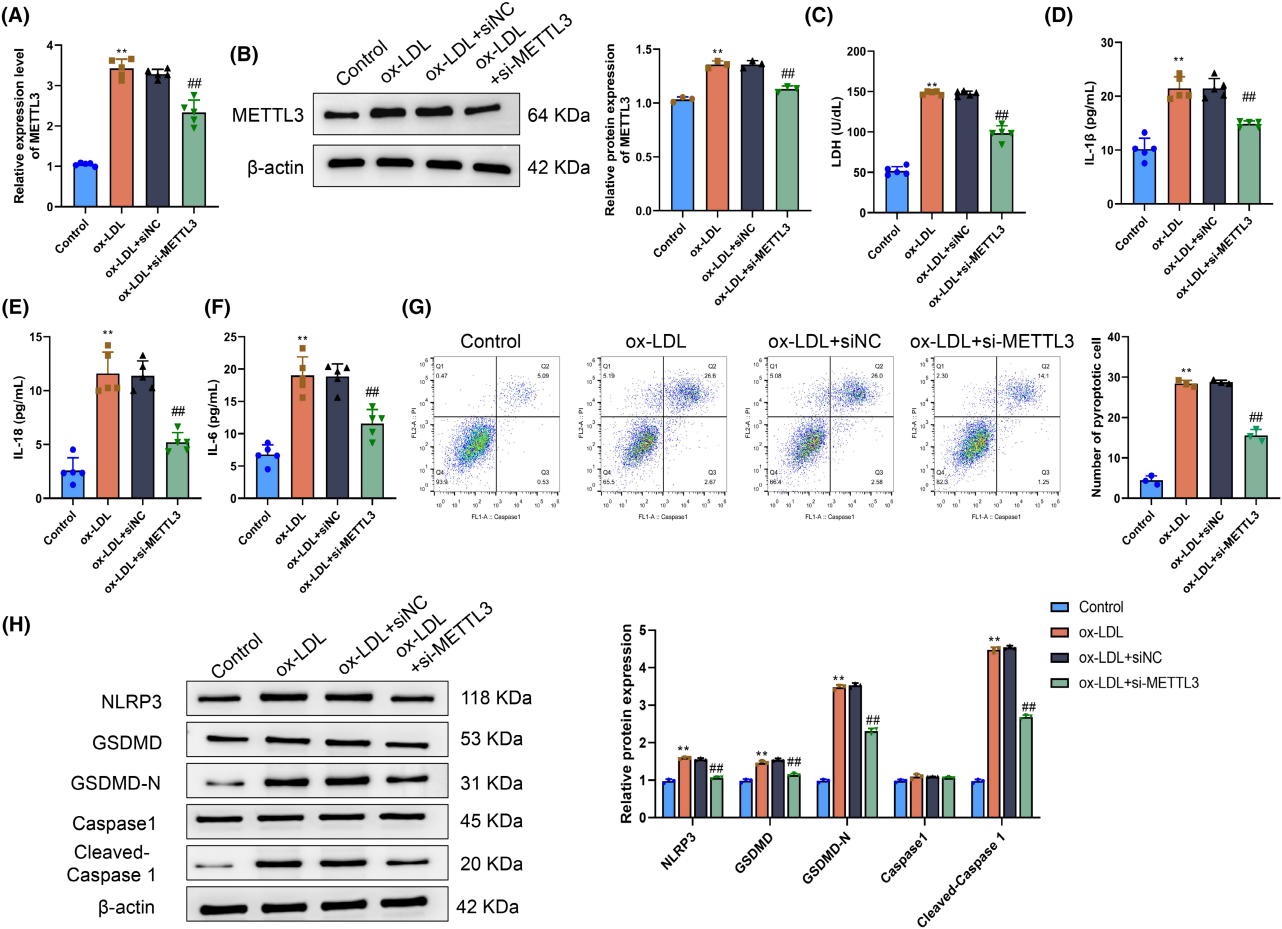
Methyltransferase‐like 3 (METTL3) knockdown inhibits pyroptosis of human aortic endothelial cells (HAECs) treated with ox‐LDL. (A) METTL3 level in HAECs in the Control, ox‐LDL, ox‐LDL+siNC, and ox‐LDL+si‐METTL3 groups determined by RT‐qPCR. (B) METTL3 protein level in HAECs in the Control, ox‐LDL, ox‐LDL+siNC, and ox‐LDL+si‐METTL3 groups determined using western blotting. (C) Lactate dehydrogenase (LDH) levels in the supernatant of HAECs in the Control, ox‐LDL, ox‐LDL+siNC, and ox‐LDL+si‐METTL3 groups. (D–F) Levels of IL‐1β (D), IL‐18 (E), and IL‐6 (F) in the cell culture supernatants of HAECs in the Control, ox‐LDL, ox‐LDL+siNC, and ox‐LDL+si‐METTL3 groups determined using ELISA. (G) The FLICA 660 Caspase‐1 assay was used to determine the level of pyroptosis in the Control, ox‐LDL, ox‐LDL+siNC, and ox‐LDL+si‐METTL3 groups. (H) Western blotting was used to assess the expression levels of proteins associated with pyroptosis (NLRP3, GSDMD, GSDMD‐N, caspase‐1, and cleaved caspase‐1) in human aortic endothelial cells (HAECs) in the Control, ox‐LDL, ox‐LDL+siNC, and ox‐LDL+si‐METTL3 groups. **p < .01 versus Control group; ^##^ *p* < .01 versus ox‐LDL+siNC group.

After discovering that METTL3 was highly expressed in both the animal and cell models of atherosclerosis, we hypothesized that the level of METTL3 expression might affect HAEC function. To verify our hypothesis, we knocked down METTL3 expression using si‐METTL3 in HAECs. The RT‐qPCR and western blotting results showed that the levels of METTL3 mRNA and protein in HAECs in the ox‐LDL group were significantly increased compared with those in HAECs in the Control group (*p* < .05). The METTL3 mRNA and protein levels in HAECs in the ox‐LDL+si‐METTL3 group were moderately decreased compared with those in the ox‐LDL+siNC group (*p* < .05). The differences in METTL3 mRNA and protein levels between the ox‐LDL and ox‐LDL+siNC groups were not statistically significant (*p* > .05) (Figure 2A,B). These results demonstrated the successful knockdown of METTL3 by transfection with si‐METTL3. Subsequently, we investigated whether METTL3 knockdown affected the cell survival rate. There was a notable rise in LDH levels in the cell culture supernatant of the ox‐LDL group, while a marked reduction in extracellular LDH levels of HAECs was observed in the culture supernatant of the ox‐LDL+si‐METTL3 group compared with the ox‐LDL+siNC group (*p* < .05) (Figure 2C). We also hypothesized the concentrations of extracellular inflammatory markers IL‐1β, IL‐18, and IL‐6 using ELISA. There were notable elevations in the levels of IL‐1β, IL‐18, and IL‐6 in the supernatant of HAECs in the ox‐LDL group compared with the levels in the Control group. Conversely, the levels of inflammatory cytokines secreted by HAECs in the ox‐LDL+si‐METTL3 group were significantly reduced compared with the levels in the ox‐LDL+siNC group (*p* < .05) (Figure 2D–F). These findings indicated a notable elevation in the level of pyroptosis of HAECs in the ox‐LDL group compared with the level of pyroptosis of HAECs in the Control group. Conversely, the level of pyroptosis of HAECs in the ox‐LDL+si‐METTL3 group was significantly decreased compared with the level of pyroptosis of HAECs in the ox‐LDL+siNC group (*p* < .05) (Figure 2G). At the same time, we hypothesized the levels of pyroptosis‐associated proteins NLRP3, GSDMD, GSDMD‐N, caspase‐1, and cleaved caspase‐1. Compared with the HAECs in the ox‐LDL+siNC group, the levels of NLRP3, GSDMD, GSDMD‐N, and cleaved caspase‐1 in HAECs in the ox‐LDL group were elevated. Conversely, there was a notable decrease in the expression of pyroptosis‐related proteins in HAECs in the ox‐LDL+si‐METTL3 group compared with the expression of pyroptosis‐related proteins in the ox‐LDL+siNC group (*p* < .05) (Figure 2H).

### 3.3 Changes in METTL3 expression affect the monocyte adhesion ability of HAECs treated with ox‐LDL

**FIGURE 3:**
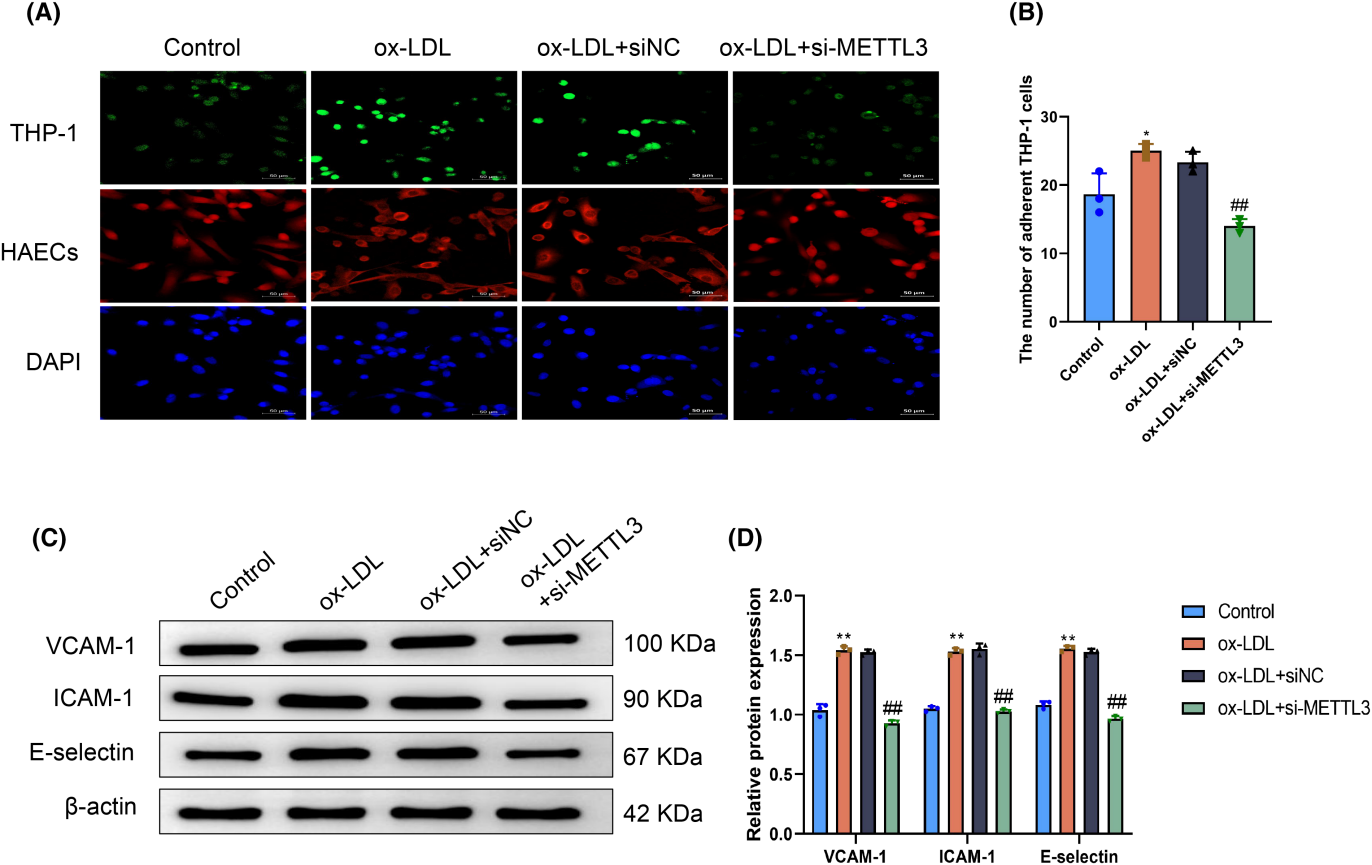
Methyltransferase‐like 3 (METTL3) knockdown inhibits monocyte adhesion ability of ox‐LDL‐treated human aortic endothelial cells (HAECs). (A) THP‐1 cells were labeled with a fluorescent dye and then cocultured with HAECs in the Control, ox‐LDL, ox‐LDL+siNC, and ox‐LDL+si‐METTL3 groups, respectively. Monocyte adhesion was detected by immunofluorescence. (B) Quantitative analysis results of the number of adhered THP‐1 cells. (C) Protein levels of VCAM‐1, ICAM‐1, and E‐selectin in HAECs in the Control, ox‐LDL, ox‐LDL+siNC, and ox‐LDL+si‐METTL3 groups. (D) Quantitative analysis results of the protein levels of VCAM‐1, ICAM‐1, and E‐selectin. *p < .05, **p < .01 versus Control group; ^##^ *p* < .01 versus ox‐LDL+siNC group.

The adhesion of monocytes and endothelial cells in great vessels is a key pathological event in the occurrence and progression of atherosclerosis.^29^ Therefore, we evaluated the influence of METTL3 expression levels on the monocyte adhesion ability of HAECs. Immunofluorescence results showed that the number of THP‐1 cells that adhered to HAECs in the ox‐LDL group was significantly increased compared with the number of THP‐1 cells that adhered to HAECs in the Control group (*p* < .05). In contrast, the number of THP‐1 cells adhered to HAECs in the ox‐LDL+si‐METTL3 group was markedly decreased compared with the number of THP‐1 cells adhered to HAECs in the ox‐LDL+siNC group (*p* < .05). There was no significant difference between the number of THP‐1 cells adhered to HAECs in the ox‐LDL and ox‐LDL+siNC groups (*p* > .05) (Figure 3A,B). We also compared differences in the protein levels of VCAM‐1, ICAM‐1, and E‐selectin in HAECs in each group. These proteins are crucial for cell adhesion because they enhance the interaction between endothelial cells and monocytes and influence the location and adherence of monocytes to blood vessel walls.^30^ Western blotting results revealed that compared with the Control group, the protein levels of VCAM‐1, ICAM‐1, and E‐selectin in HAECs in the ox‐LDL group increased significantly (*p* < .05). Compared with the ox‐LDL+siNC group, the protein levels of VCAM‐1, ICAM‐1, and E‐selectin in HAECs in the ox‐LDL+si‐METTL3 group were markedly decreased (*p* < .05). The protein levels of VCAM‐1, ICAM‐1, and E‐selectin in the ox‐LDL and ox‐LDL+siNC groups did not differ significantly (*p* > .05) (Figure 3C,D). The above results demonstrated that METTL3 expression levels significantly affected the monocyte adhesion ability of HAECs treated with ox‐LDL, and METTL3 knockdown markedly inhibited the interaction between HAECs treated with ox‐LDL and monocytes.

### 3.4 METTL3 enhances H19 stability through m6A modification, thereby regulating H19 expression

**FIGURE 4:**
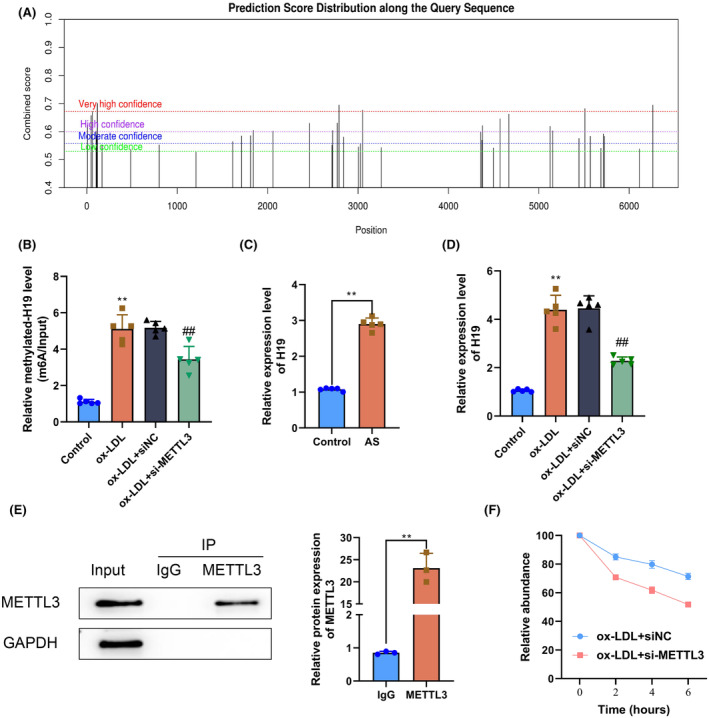
Methyltransferase‐like 3 (METTL3)‐mediated N^6^‐methyladenosine (m6A) modification enhances H19 stability to regulate H19 expression. (A) Potential m6A modification sites in the lncRNA H19 sequence as predicted by the SRAMP database. (B) M6A abundance level of H19 in human aortic endothelial cells (HAECs) in the Control, ox‐LDL, ox‐LDL+siNC, and ox‐LDL+si‐METTL3 groups determined by MeRIP‐qPCR. (C) H19 expression levels in the aortas of mice in the Control and AS groups determined by RT‐qPCR. (D) H19 expression levels in HAECs in the Control, ox‐LDL, ox‐LDL+siNC, and ox‐LDL+si‐METTL3 groups determined by RT‐qPCR. (E) RIP experiment to determine whether H19 blinds to METTL3 in HAECs. (F) HAECs were treated with actinomycin D in the RNA stability experiment. The residual H19 levels in the ox‐LDL+siNC and ox‐LDL+si‐METTL3 groups were determined using RT‐qPCR at specific time points. **p < .01 versus Control group; ^##^ *p* < .01 versus ox‐LDL+siNC group.

H19 is closely related to atherosclerosis progression.^31^ To clarify whether H19 was involved in the process of METTL3 aggravating atherosclerosis, we first consulted the SRAMP database and learned that H19 had as many as 41 potential m6A modification sites (Figure 4A). Such a finding preliminarily suggested the huge likelihood of H19 accepting METTL3‐mediated m6A modification. Therefore, we further investigated whether METTL3 affected H19 by directly mediating its m6A modification. MeRIP‐qPCR results showed that compared with the Control group, the m6A abundance level of H19 in HAECs in the ox‐LDL group was significantly increased (*p* < .05). Compared with the ox‐LDL+siNC group, the m6A abundance level of H19 in HAECs in the ox‐LDL+si‐METTL3 group was markedly decreased (*p* < .05). The difference in the m6A abundance level of H19 in HAECs in the ox‐LDL and ox‐LDL+siNC groups was not significant (*p* > .05) (Figure 4B). RT‐qPCR of mouse aorta tissues showed that compared with the Control group, the H19 expression level in the aortas of mice in the atherosclerosis group was significantly increased (*p* < .05) (Figure 4C). Similarly, the RT‐qPCR results indicated that compared with the Control group, H19 expression levels in HAECs in the ox‐LDL group were markedly increased. Compared with the ox‐LDL+siNC group, H19 expression in HAECs in the ox‐LDL+si‐METTL3 group was notably decreased (*p* < .05). The difference in H19 expression levels in HAECs between the ox‐LDL and ox‐LDL+siNC groups was not statistically significant (*p* > .05) (Figure 4D). Additionally, the RIP experiment result suggested that H19 in HAECs bound to METTL3 (Figure 4E). Therefore, METTL3 regulated the expression level of H19 by direct m6A modification in both the animal and cell models of atherosclerosis. Thus, we further explored the regulatory mechanism of METTL3‐mediated H19 expression by treating HAECs in each group with actinomycin D. Briefly, before actinomycin D exposure, the percentages of the residual H19 level in HAECs in the ox‐LDL+siNC and ox‐LDL+si‐METTL3 groups showed no significant difference (*p* > .05). Following actinomycin D exposure for 2, 4, and 6 h, the percentage of residual H19 levels in HAECs in the ox‐LDL+si‐METTL3 group was markedly decreased compared with the percentage of residual H19 levels in HAECs in the ox‐LDL+siNC group (*p* < .05) (Figure 4F). Thus, METTL3 improved H19 stability by m6A modification. Collectively, the above results indicated that METTL3 enhanced H19 stability through m6A modification, thereby regulating the level of H19 expression.

### 3.5 IGF2BP2 binds to H19 to enhance its stability and regulate its expression

**FIGURE 5:**
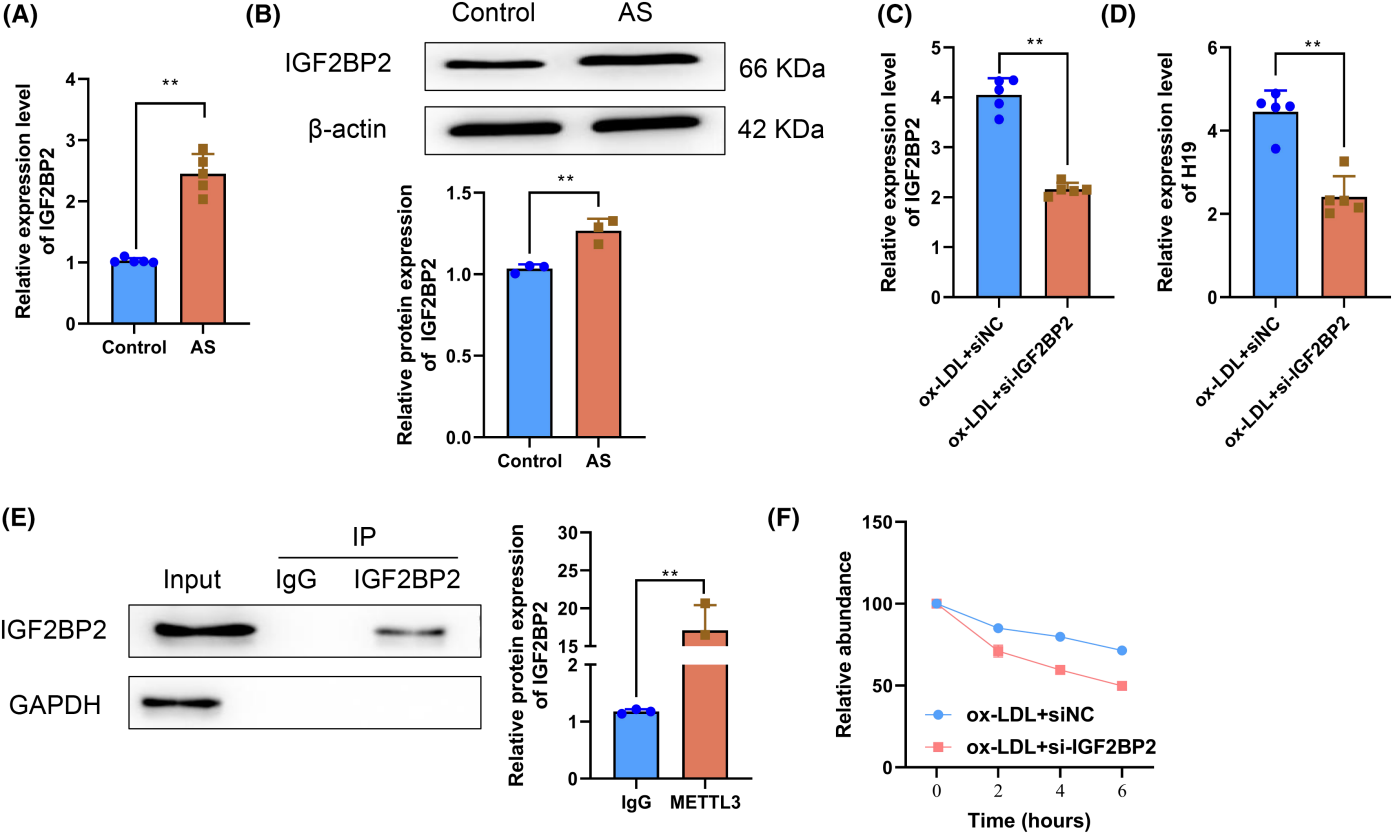
IGF2BP2 binds to H19 to enhance its stability and regulate its expression. (A) IGF2BP2 mRNA expression levels in the aortas of mice in the Control and AS groups determined by RT‐qPCR. (B) IGF2BP2 protein levels in the aortas of mice in the Control and AS groups determined by western blotting. (C) IGF2BP2 mRNA expression levels in human aortic endothelial cells (HAECs) in the ox‐LDL+siNC and ox‐LDL+si‐IGF2BP2 groups determined by RT‐qPCR. (D) H19 expression levels in HAECs in the ox‐LDL+siNC and ox‐LDL+si‐IGF2BP2 groups determined by RT‐qPCR. (E) RIP experiment to determine whether H19 binds to IGF2BP2 in HAECs. (F) In the RNA stability experiment, HAECs were treated with actinomycin D, and the residual levels of H19 in the ox‐LDL+siNC and ox‐LDL+si‐IGF2BP2 groups were determined using RT‐qPCR at different time points. **p < .01 versus Control group.

In m6A modification, the writer and reader proteins affect the stability and expression level of the target RNA molecules.^32^ Because we had discovered that writer METTL3 affected H19 expression by regulating its stability, we further investigated whether the reader IGF2BP2 also had the same function. RT‐qPCR and western blotting results indicated that compared with the Control group, IGF2BP2 mRNA and protein levels in the aortas of mice in the atherosclerosis group were significantly increased (*p* < .05) (Figure 5A,B). Subsequently, si‐IGF2BP2 was used to knock down the expression of IGF2BP2 in HAECs to observe the influence of IGF2BP2 on H19 expression. RT‐qPCR results showed that compared with the ox‐LDL+siNC group, IGF2BP2 mRNA levels in HAECs in the ox‐LDL+si‐IGF2BP2 group were significantly decreased (*p* < .05) (Figure 5C), confirming the successful knockdown of IGF2BP2 by si‐IGF2BP2 transfection. Furthermore, compared with the ox‐LDL+siNC group, the H19 expression level in HAECs in the ox‐LDL+si‐IGF2BP2 group was markedly decreased (*p* < .05) (Figure 5D). Moreover, the RIP experiment result also indicated that H19 might bind to IGF2BP2 in HAECs (Figure 5E). Therefore, binding of the reader IGF2BP2 to H19 regulated its expression in the animal and cell models of atherosclerosis. Then, we further explored the regulatory mechanism of IGF2BP2‐mediated H19 expression by treating HAECs in each group with actinomycin D. Specifically, before exposure to actinomycin D, the percentages of residual H19 in HAECs in the ox‐LDL+siNC and si‐IGF2BP2 groups were not significantly different (*p* > .05). Following exposure to actinomycin D for 2, 4, and 6 h, the percentage of residual H19 levels in HAECs in the ox‐LDL+si‐IGF2BP2 group was markedly decreased compared with the percentage of residual H19 levels in HAECs in the ox‐LDL+siNC group (*p* < .05) (Figure 5F). Such findings indicated that IGF2BP2 also regulated the stability of H19. Collectively, the above results suggested that m6A modification‐related reader IGF2BP2 bound to H19 to improve its stability and regulate its expression.

### 3.6 METTL3 promotes HAEC pyroptosis by regulating H19

**FIGURE 6:**
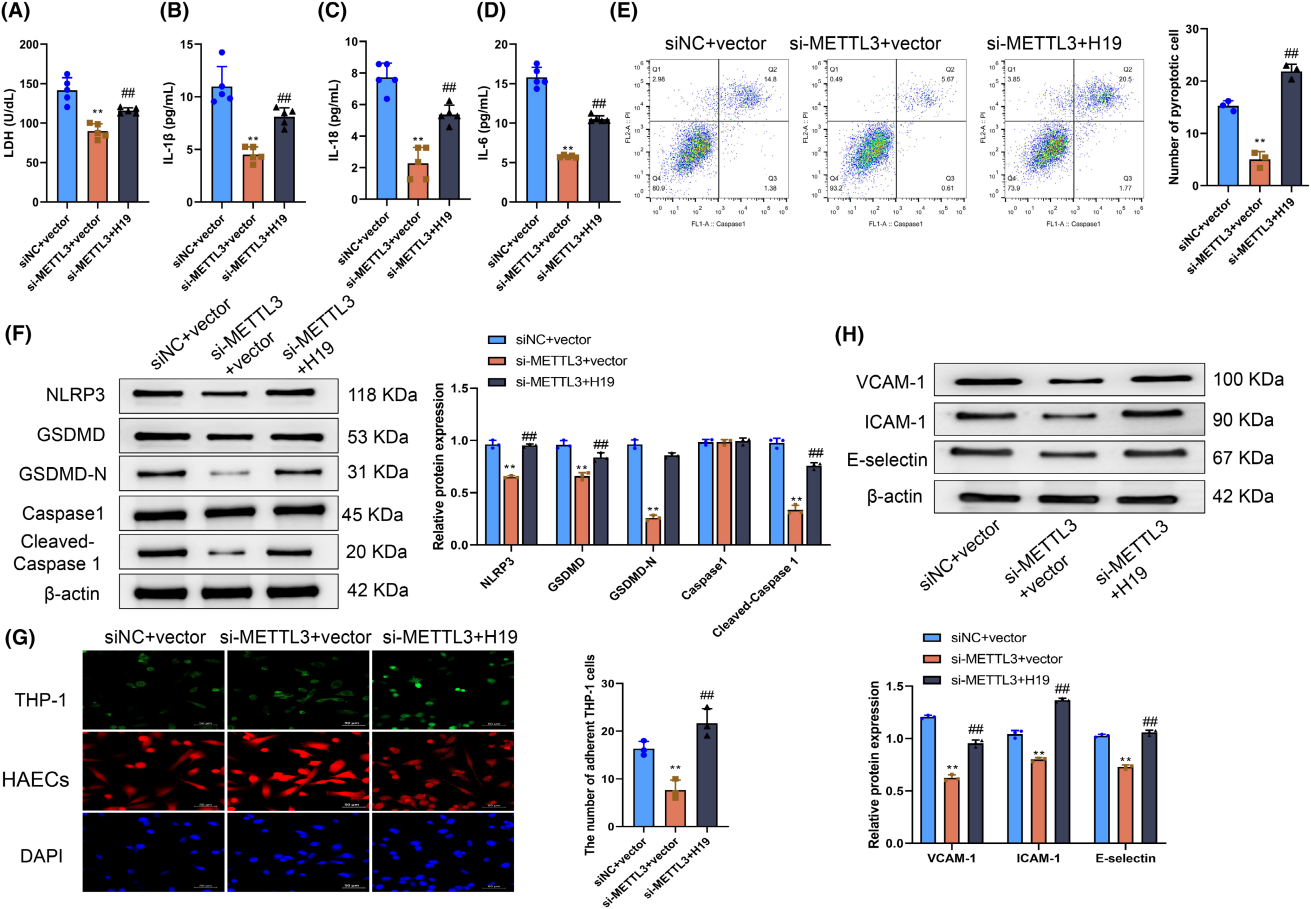
Methyltransferase‐like 3 (METTL3) promotes human aortic endothelial cells (HAEC) pyroptosis by upregulating H19. (A) Lactate dehydrogenase (LDH) levels in the supernatant of HAECs measured in the si‐METTL3+vector and si‐METTL3+H19 groups. (B–D) ELISA was used to determine the levels of IL‐1β (B), IL‐18 (C), and IL‐6 (D) in the cell culture supernatants of HAECs in the siNC+vector, si‐METTL3+vector, and si‐METTL3+H19 groups. (E) Pyroptosis of HAECs in siNC+vector, si‐METTL3+vector, and si‐METTL3+H19 groups determined using flow cytometry. (F) Levels of NLRP3, GSDMD, GSDMD‐N, caspase‐1, and cleaved caspase‐1 in HAECs in the siNC+vector, si‐METTL3+vector, and si‐METTL3+H19 groups determined using western blotting. (G) THP‐1 cells were labeled with a fluorescent dye and then cocultured with HAECs in the siNC+vector, si‐METTL3+vector, and si‐METTL3+H19 groups, respectively. Monocyte adhesion was detected by immunofluorescence. (H) Protein levels of VCAM‐1, ICAM‐1, and E‐selectin in HAECs of the siNC+vector, si‐METTL3+vector, and si‐METTL3+H19 groups determined using western blotting. **p < .01 versus siNC+vector group; ^##^ *p* < .01 versus si‐METTL3+vector group.

Huang et al. pointed out that the H19 overexpression was a key link in the occurrence of atherosclerosis.^33^ It was already established that METTL3 affected multiple biological behaviors of HAECs and regulated H19 expression. Therefore, we further investigated whether the mechanism by which METTL3 affected HAEC pyroptosis was related to its regulation of H19 expression. Initially, we investigated extracellular LDH activity under various treatments, revealing a notable elevation in the LDH levels in the cell culture supernatant of HAECs in the si‐METTL3+H19 group compared with the LDH levels in the cell culture supernatant of HAECs in the si‐METTL3+vector group (*p* < .05) (Figure 6A). While assessing the secretion levels of various factors within the cells, we concurrently observed that the concentrations of IL‐1β, IL‐18, and IL‐6 in the cell culture supernatant of HAECs in the si‐METTL3+H19 experimental group exhibited a notable increase compared with the concentrations of IL‐1β, IL‐18, and IL‐6 in the si‐METTL3+vector control group (*p* < .05) (Figure 6B–D). The flow cytometry analysis results revealed a notable elevation in the pyroptosis level of HAECs in the si‐METTL3+H19 group compared with HAECs in the si‐METTL3+vector group (*p* < .05) (Figure 6E). Concurrently, western blotting revealed that the protein expression levels of NLRP3, GSDMD, GSDMD‐N, and cleaved caspase‐1 in HAECs were notably elevated in the si‐METTL3+H19 group compared with those in the si‐METTL3+vector group (*p* < .05) (Figure 6F). Immunofluorescence results showed that compared with the siNC+vector group, the number of THP‐1 cells adhered to HAECs in the si‐METTL3+vector group was significantly decreased (*p* < .05). The number of THP‐1 cells adhered to HAECs in the si‐METTL3+H19 group was markedly increased compared with the si‐METTL3+vector group (*p* < .05) (Figure 6G). Additionally, the western blotting results suggested that compared with the siNC+vector group, the protein levels of VCAM‐1, ICAM‐1, and E‐selectin in HAECs of the si‐METTL3+vector group were significantly decreased (*p* < .05). Compared with HAECs in the si‐METTL3+vector group, the protein levels of VCAM‐1, ICAM‐1, and E‐selectin in HAECs of the si‐METTL3+H19 group were significantly increased (*p* < .05) (Figure 6H). The above results demonstrated that H19 overexpression effectively enhanced the pyroptosis of HAECs decreased due to METTL3 knockdown. In other words, METTL3 was extremely likely to improve the pyroptosis of HAECs by upregulating H19 expression.

### 3.7 METTL3 promotes atherosclerosis progression by regulating H19 expression in vivo

**FIGURE 7:**
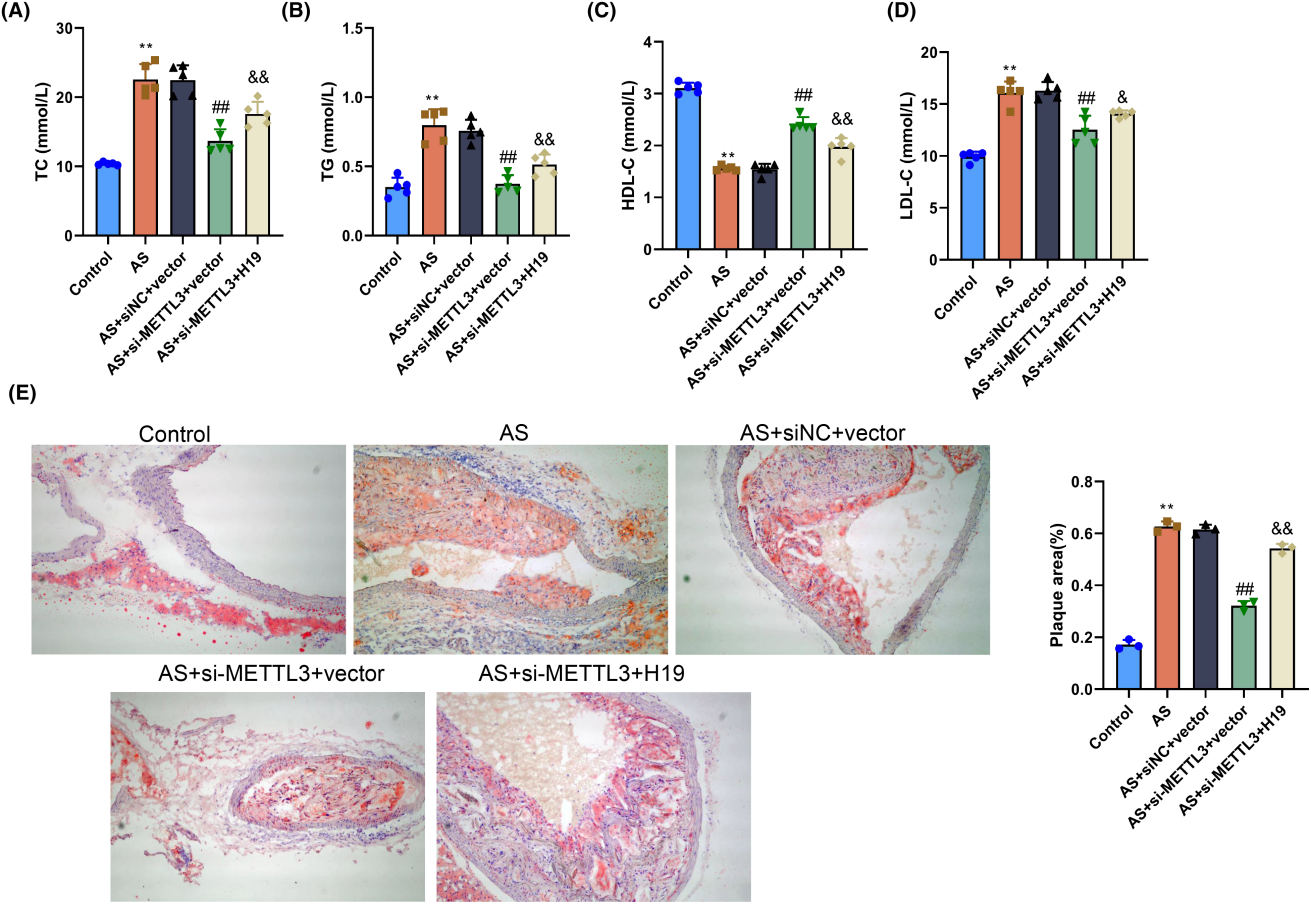
Methyltransferase‐like 3 (METTL3) promotes AS progression by regulating H19 expression in vivo. (A–D) Serum total cholesterol (TC), triglycerides (TG), low‐density lipoprotein cholesterol (LDL‐C), levels in mice in the Control, AS, AS+siNC+vector, AS+si‐METTL3+vector, and AS+si‐METTL3+H19 groups determined by ELISA. (E) Plaque formation in the aortas of mice in the Control, AS, AS+siNC+vector, AS+si‐METTL3+vector, and AS+si‐METTL3+H19 groups was observed using oil red O staining, and the ratio of plaque to the vascular area was quantitatively analyzed. **p < .01 versus Control group; ^##^ *p* < .01 versus AS+siNC+vector group; ^&&^ *p* < .01 versus AS+si‐METTL3+vector group.

To study the effect and potential mechanism of METTL3 on atherosclerosis in vivo, lentivirus containing si‐METTL3 and pcDNA3.1‐H19 was introduced into mice by tail vein injection to knock down METTL3 and overexpress H19, respectively. Subsequently, RT‐qPCR and western blot experiments confirmed that si‐METTL3 significantly reduced the mRNA and protein expression of METTL3 in the aorta of AS model mice (*p* < .05, Figure S1A,B). ELISA results demonstrated that compared with the Control group, serum TC, TG, and LDL‐C levels in mice in the atherosclerosis group were significantly increased and HDL‐C was significantly decreased (*p* < .05). Compared with the atherosclerosis+siNC+vector group, serum TC, TG, and LDL‐C levels in mice in the atherosclerosis+si‐METTL3+vector group significantly decreased (*p* < .05), while the HDL‐C level markedly increased. In contrast with the atherosclerosis+si‐METTL3+vector group, serum TC, TG, and LDL‐C levels in mice in the atherosclerosis+si‐METTL3+H19 group significantly increased (*p* < .05), while the HDL‐C level markedly decreased. There was no statistically significant difference between the atherosclerosis and atherosclerosis+siNC+vector groups (*p* > .05) (Figure 7A–D).

Oil red O staining revealed that the aortas of mice in the Control group were smooth and flat, without the formation of visible and obvious red fat plaques. Plaques of varying degrees were observed in the aortas of mice in the atherosclerosis, atherosclerosis+siNC+vector, atherosclerosis+si‐METTL3+vector, and atherosclerosis+si‐METTL3+H19 groups, and they were distributed across various sites. Moreover, compared with the Control group, the ratio of plaque to the vascular area in the atherosclerosis group was significantly increased (*p* < .05). Compared with the atherosclerosis+siNC+vector group, the ratio of plaque to the vascular area in the aortas of mice in the atherosclerosis+si‐METTL3+vector group was markedly decreased (*p* < .05). Compared with the atherosclerosis+si‐METTL3+vector group, the ratio of plaque to the vascular area in the aortas of mice in the atherosclerosis+si‐METTL3+H19 group was significantly increased (*p* < .05). The difference between the atherosclerosis and atherosclerosis+siNC+vector groups was not statistically significant (*p* > .05) (Figure 7E). These results demonstrated that METTL3 promoted atherosclerosis progression by regulating H19 expression in the mouse model.

In a nutshell, the above results collectively illustrated that METTL3 regulation of H19 expression promoted atherosclerosis progression in vivo.

### 3.8 METTL3 inhibits pyroptosis by regulating H19 expression in vivo

**FIGURE 8:**
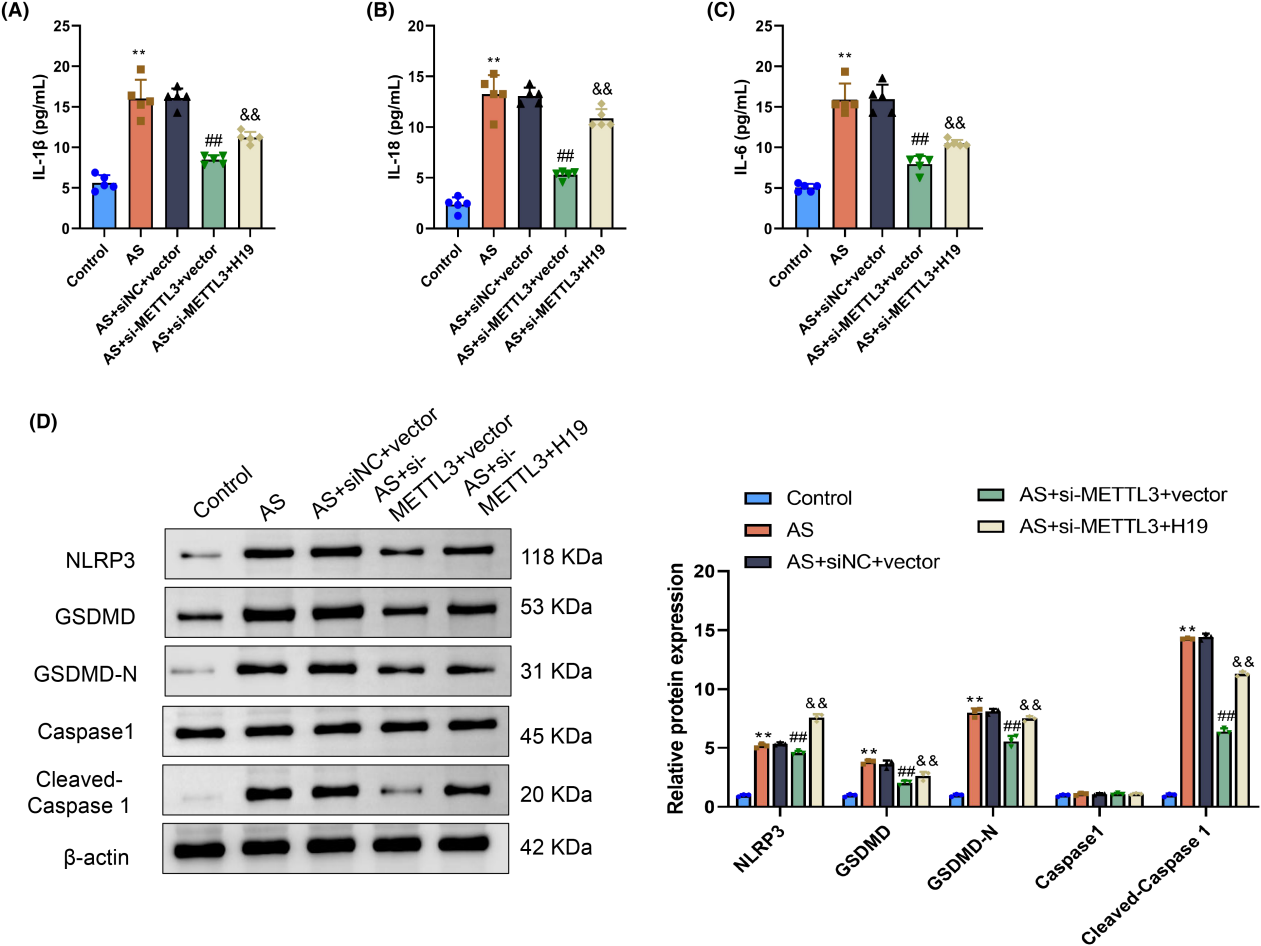
Methyltransferase‐like 3 (METTL3) inhibits pyroptosis by regulating H19 expression in vivo. (A–C) Serum levels of IL‐1β, IL‐18, and IL‐6 in mice in the Control, AS, AS+siNC+vector, AS+si‐METTL3+vector, and AS+si‐METTL3+H19 groups determined by ELISA. (D) Protein expression levels of NLRP3, GSDMD, GSDMD‐N, caspase‐1, and cleaved caspase‐1 in the Control, AS, AS+siNC+vector, AS+si‐METTL3+vector, and AS+si‐METTL3+H19 groups determined by western blotting. **p < .01 versus Control group; ^##^ *p* < .01 versus AS+siNC+vector group; ^&&^ *p* < .01 versus AS+si‐METTL3+vector group.

The aforementioned studies illustrated the impact of METTL3 and H19 on animal models to elucidate the correlation between METTL3 and H19 in vivo and pyroptosis levels. A notable increase in serum IL‐1β, IL‐18, and IL‐6 levels was observed in atherosclerosis mice, and METTL3 knockdown in atherosclerosis mice significantly decreased these inflammatory markers. Conversely, upon H19 overexpression subsequent to METTL3 knockdown, the serum levels of IL‐1β, IL‐18, and IL‐6 were significantly elevated relative to the METTL3 knockdown condition (*p* > .05) (Figure 8A–C). Following our examination of the thoracic aorta samples, we noted that proteins linked to pyroptosis, such as NLRP3, GSDMD, GSDMD‐N, and caspase‐1, exhibited altered expression levels. Specifically, there was a notable increase in the expression of cleaved caspase‐1. However, the expression levels of these proteins decreased significantly upon METTL3 knockdown. Furthermore, the introduction of H19 after METTL3 knockdown markedly elevated the expression levels of NLRP3, GSDMD, GSDMD‐N, caspase‐1, and cleaved caspase‐1 compared with METTL3 knockdown alone (*p* > .05) (Figure 8D). The above findings indicate that METTL3 modulates the pyroptosis status of blood vessels by suppressing lncRNA H19.

## 4 DISCUSSION

Our results showed that the expression levels of m6A, METTL3, and METTL14 were significantly increased in cell and animal models of atherosclerosis. But the relative increase in METTL3 and METTL14 at the protein level is much more moderate compared with the increase at the mRNA level. The reasons may due to Gene expression is not solely controlled at the transcriptional level; post‐transcriptional mechanisms such as mRNA splicing, transport, stability, and translation efficiency can all influence the final protein expression levels. Protein levels depend not only on synthesis rates but also on degradation rates. Cellular pathways like the ubiquitin‐proteasome system and lysosomal degradation can accelerate the breakdown of certain proteins, resulting in a less pronounced increase at the protein level compared with mRNA. The moderate knockdown efficiency of si‐METTL3 could be attributed to several factors, such as siRNA molecules may be unstable within cells and prone to degradation by intracellular nucleases, which can reduce the effective concentration of siRNA and impair knockdown efficiency. Even if siRNA successfully reduces mRNA levels, cells may employ other post‐transcriptional regulatory mechanisms (such as alternative splicing or mRNA stability regulation) to maintain a certain level of METTL3 expression. Additionally, METTL3 knockdown weakened HAEC pyroptosis in vitro. Furthermore, H19 was identified as the main target lncRNA molecule of m6A modification, and the writer METTL3 and reader IGF2BP2 upregulated H19 expression by enhancing H19 stability. Moreover, the H19 that was upregulated by m6A modification was likely to aggravate atherosclerosis. Finally, in vivo experiments further verified the influence of METTL3‐mediated m6A modification and H19 expression in atherosclerosis. To our knowledge, this study is the first to reveal that m6A modification aggravated atherosclerosis by regulating an lncRNA molecule. The clinical significance of this study lies in the identification of a novel target for the treatment of atherosclerosis. In other words, inhibiting the m6A modification process of H19 may be a new treatment strategy.

In this study, we first compared the differences in m6A levels and relevant writer protein expression levels between the experimental and control models of atherosclerosis. The results showed that m6A, METTL3, and METTL14 expression levels in the atherosclerosis experimental models were much higher than those in the control models. As reported in a meta‐analysis,^34^ the expression levels of METTL3 and METTL14 were increased in vascular endothelial cell, macrophage, and smooth muscle cell models of atherosclerosis, consistent with our study findings.

We also observed the impact of METTL3 knockdown on the biological behavior of the atherosclerosis cell model, particularly focusing on HAEC pyroptosis, given the critical role of endothelial cell dysfunction in the pathogenesis of atherosclerosis.^35^ Specifically, during the initial phases of atherosclerosis, endothelial cell pyroptosis compromises cellular integrity, resulting in the discharge of inflammatory cytokines and heightened vascular permeability, as well as attracting monocytes.^7^ Abnormal tube formation in vascular endothelial cells contributes to excessive thickening of the vascular inner wall, thereby increasing the risk of atheromatous plaque formation. Moreover, when the monocyte adhesion ability of vascular endothelial cells is excessively enhanced, massive amounts of monocytes are recruited to the inner wall of blood vessels. These monocytes are transformed into macrophages that phagocytose lipids, such as TC, TG, and LDL‐C, forming the core components of atherosclerotic plaques.^36^ Our findings showed that METTL3 knockdown significantly inhibited HAEC pyroptosis, illustrating in reverse that METTL3‐mediated m6A modification is a key aggravating factor of atherosclerosis. This conclusion is basically in line with the findings of Zhang et al. and Liu et al.^20^, ^21^

After preliminarily verifying that m6A modification promoted HAEC pyroptosis in atherosclerosis, we identified the main lncRNA molecule affected by m6A modification as H19. A significant correlation between H19 and pyroptosis has already been reported.^37^ Liu et al. proposed that H19 had the potential to induce inflammasome activation, leading to pyroptosis within the arachnoid membrane of rats.^38^ Guo et al. demonstrated that H19 had the capability to induce pyroptosis in neuronal cells.^39^ However, other studies reported an inverse relationship between H19 and the incidence of pyroptosis. Han et al. proposed that H19 suppressed cardiomyocyte pyroptosis by acting on PBX3/CYP1B1.^40^ Yang et al. noted that H19 suppressed pyroptosis in fibroblasts.^41^ Thus, the controversy surrounding the association between H19 and pyroptosis prompted us to focus on H19 in our investigation to elucidate the mechanism of atherosclerosis induction by METTL3. Additionally, clinical studies have proved that the H19 expression level in the peripheral blood of patients with atherosclerosis was obviously higher than that of ordinary subjects.^15^, ^42^ Basic studies have also shown that H19 promotes atherosclerosis progression by promoting excessive vascular endothelial cell proliferation,^43^ inhibiting adipocyte differentiation,^44^ and inducing lipid metabolism disorders.^45^ Conversely, bioinformatics data in our study revealed that H19 had as many as 41 possible m6A modification sites, among which 13 were sites of high confidence sites and six were sites of very high confidence, demonstrating that H19 was a possible major lncRNA molecule modified by m6A in the pathological process of atherosclerosis. According to the findings of these aforementioned studies, we inferred that H19 might be a pivotal element in the mechanism by which METTL3 induces atherosclerosis. Subsequently, we confirmed our assumption by relevant analysis of METTL3 and H19 expression levels and an RIP experiment, verifying the role of H19 in vivo and in vitro. Briefly, H19 overexpression significantly aggravated atherosclerosis alleviated by METTL3 knockdown. Thus, METTL3 promoted atherosclerosis progression by upregulating H19 expression levels in vivo and in vitro.

It was reported that after m6A modification of RNA molecules through the mediation of the writer METTL3, subsequent identification required the help of a reader protein.^46^ Among the m6A modification‐related reader proteins, cytoplasmic protein IGF2BP2 was proven to identify and directly bind to m6A sites via two unique K homology domains contained at its end. After binding to the RNA molecule, IGF2BP2 prevents RNA degradation enzymes from binding to the target RNA to increase the stability of the RNA molecule, direct RNA molecules to translation complexes to accelerate protein synthesis, and promote RNA aggregation at specific subcellular locations.^47^ On the basis of the important role of reader protein IGF2BP2 in regulating RNA stability, translation, and transport, this study also explored whether IGF2BP2 had a similar effect on H19. We found that reader protein IGF2BP2 bound to H19 to improve its stability and then upregulated H19 expression. Subsequent in vivo experiments demonstrated that reducing METTL3 expression effectively mitigated atherosclerosis progression in mice, while H19 upregulation exacerbated the condition. These findings suggest that METTL3 facilitates atherosclerosis progression through m6A methylation modification of H19.

In our study, we observed significant downstream effects such as increased inflammatory and atherosclerotic markers, even though the reduction in METTL3 expression was moderate. This suggests that alternative mechanisms, independent of METTL3, may contribute to these downstream effects. Various methyltransferases, such as METTL14 and METTL16, exist within cells. These proteins might compensate for METTL3's function when its expression is reduced, thereby continuing to influence m6A modification and downstream signaling pathways.

While our study focuses on METTL3‐mediated effects, it is essential to consider the potential for non‐METTL3‐mediated effects that might confound our results. These could arise due to off‐target effects of the siRNA, compensation by other methyltransferases, or other regulatory pathways activated by the treatment conditions. To address and minimize the impact of potential non‐METTL3‐mediated effects, we have implemented several control measures. We used multiple siRNA sequences targeting different regions of METTL3 to ensure that the observed effects are specific to METTL3 knockdown. We included non‐targeting siRNA controls to account for any off‐target effects that might arise from siRNA delivery or processing.

We confirmed the knockdown efficiency of METTL3 at both the mRNA and protein levels using qPCR and Western blotting. This validation step ensures that the observed phenotypic changes correlate with the reduction in METTL3 expression.

There are some deficiencies in this study that must be acknowledged. First, we used siRNA to knock down METTL3, and such a method does not completely eliminate the influence of METTL3 on our experimental results. To increase the reliability of the conclusion of our study, METTL3^−/−^ transgenic mice should be constructed for further research. Second, H19 has been reported to activate multiple pathways, including the H19 pathway^48^ and the Bcl‐2 pathway.^49^ Therefore, future studies should fully analyze various types of signaling pathways through which m6A modification‐mediated H19 aggravates atherosclerosis, leading to more comprehensive and accurate conclusions.

In summary, H19 is the main target lncRNA molecule of METTL3 mediated‐m6A modification in the pathological process of atherosclerosis. Additionally, the writer protein METTL3 and reader protein IGF2BP2 bind to H19 to enhance its stability. Furthermore, m6A modification‐mediated H19 markedly promotes atherosclerosis progression by activating endothelial cell pyroptosis. Collectively, this study has revealed the little‐known mechanism of m6A participating in atherosclerosis by regulating lncRNA expression and demonstrated that targeted inhibition of METTL3 and H19 is a potential treatment strategy for atherosclerosis.

## AUTHOR CONTRIBUTIONS

Xiao‐han Zhu, Sen Yang, and Huan Zeng performed the material preparation, data collection, and analysis. Feng Tang, Long‐hai Tian, and Yong‐yao Yang wrote the first draft of this manuscript. All authors contributed to the study conception and design, commented on previous versions of the manuscript, and read and approved the final manuscript.

## FUNDING INFORMATION

This study is supported by the National Natural Science Foundation of China (82260094); the Guizhou Provincial Science and Technology Foundation Project (approval number: Qiankehe Fundamentals ZK [2021] General 355); the Science and Technology Fund of Guizhou Provincial Health Commission (approval number: gzwjkj2020‐1‐065).

### DISCLOSURES

The authors declare that they have no competing interests.

## Supporting information


Data S1.

## Data Availability

The datasets used and/or analyzed during the current study are available from the corresponding author upon reasonable request. All animal experiments included in this study were approved and monitored by the Animal Ethics Committee of our Hospital, and performed in strict accordance with *Guide to the Care and Use of Laboratory Animals* to ensure animal welfare.
