# Supplementary material for: METTL3‐mediated m6A modification enhances lncRNA H19 stability to promote endothelial cell inflammation and pyroptosis to aggravate atherosclerosis
Source: FASEB J. 2024 Oct 21;38(20):e70090. doi: 10.1096/fj.202401337RR (PMC11580722; doi:10.1096/fj.202401337RR)
Supplement: Supplementary file 1 — Data S1. [file FSB2-38-e70090-s001.docx]

**SUPPLEMENTAL FIGURE LEGEND**

Figure S1 METTL3 expression in the aorta of mice with atherosclerosis (AS).

A.Relative mRNA expression levels of METTL3 in control, AS, AS+siNC, and AS+si-METTL3 groups; B. Representative Western blot images and quantification of METTL3 protein levels, normalized to β-actin, across the same groups. **P < 0.01 vs. Control group; ##P < 0.01 vs. AS+siNC group.
